# Supplementary material for: Protein kinase G inhibition preserves photoreceptor viability and function in a new mouse model for autosomal dominant retinitis pigmentosa
Source: Cell Death Dis. 2025 Jul 30;16(1):575. doi: 10.1038/s41419-025-07901-9 (PMC12311109; doi:10.1038/s41419-025-07901-9)
Supplement: Supplementary file 1 — supplemental figures and tables [file 41419_2025_7901_MOESM1_ESM.pdf]

Supplemental Figures

Figure S1:

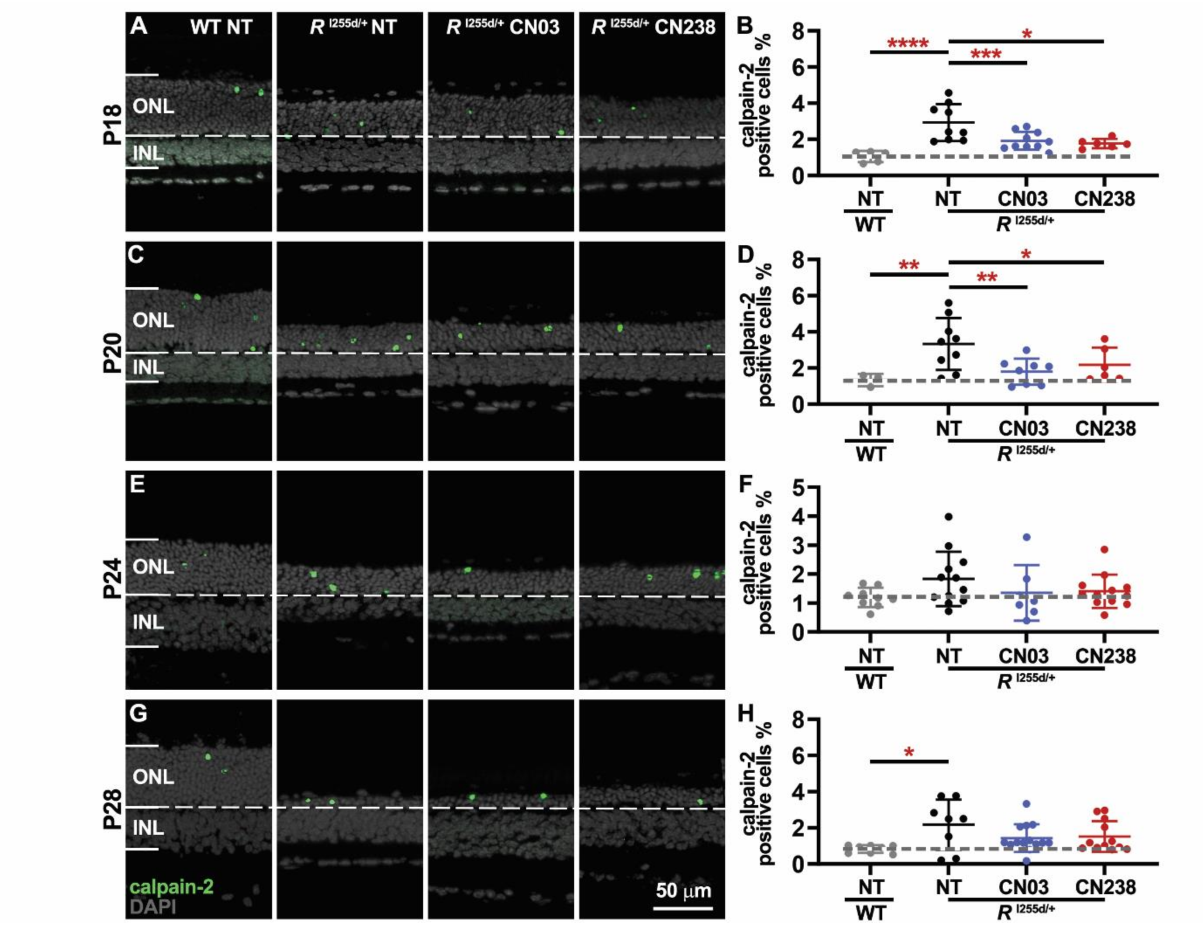

Figure S1. cGMP analogues can reduce calpain-2 activation in *Rho*$^{I255d/+}$ retina. (**A, C, E, G**) Organotypic retinal explant cultures were derived from wild-type (WT) and *Rho*$^{I255d/+}$ mice at post-natal (P) day 12, cultured without treatment for the first two days, and then treated or not with 50 µM of CN03 or CN238 for further 4, 6, 10, or 14 days *in vitro*. (**A, C, E, G**) Representative sections of retinal explants cultured until P18 (**A**), P20 (**C**), P24 (**E**), and P28 (**G**) and stained with an antibody directed against activated calpain-2 (green). DAPI (grey) was used as nuclear counterstain. Non-treated (NT) WT and *Rho*$^{I255d/+}$ cultures shown for comparison. (**B, D, F, H**) Quantification of calpain-2-positive cells in the outer nuclear layer (ONL) at P18 (**B**), P20 (**D**), P24 (**F**), and P28 (**H**). NT mutant exhibited an increased percentage of calpain-2-positive cells compared with NT WT at all time points except P24. cGMP analogues reduced calpain-2 activation in short-term cultures until P20. n = 3-14 retinas from different animals; error bars indicate SD; statistical testing: Two-way ANOVA with Dunnett's multiple comparisons test; significance level: * $p \leq 0.05$, ** $p \leq 0.01$, *** $p \leq 0.001$, **** $p \leq 0.0001$; INL = inner nuclear layer; scale bar = 50 µm.

Figure S2:

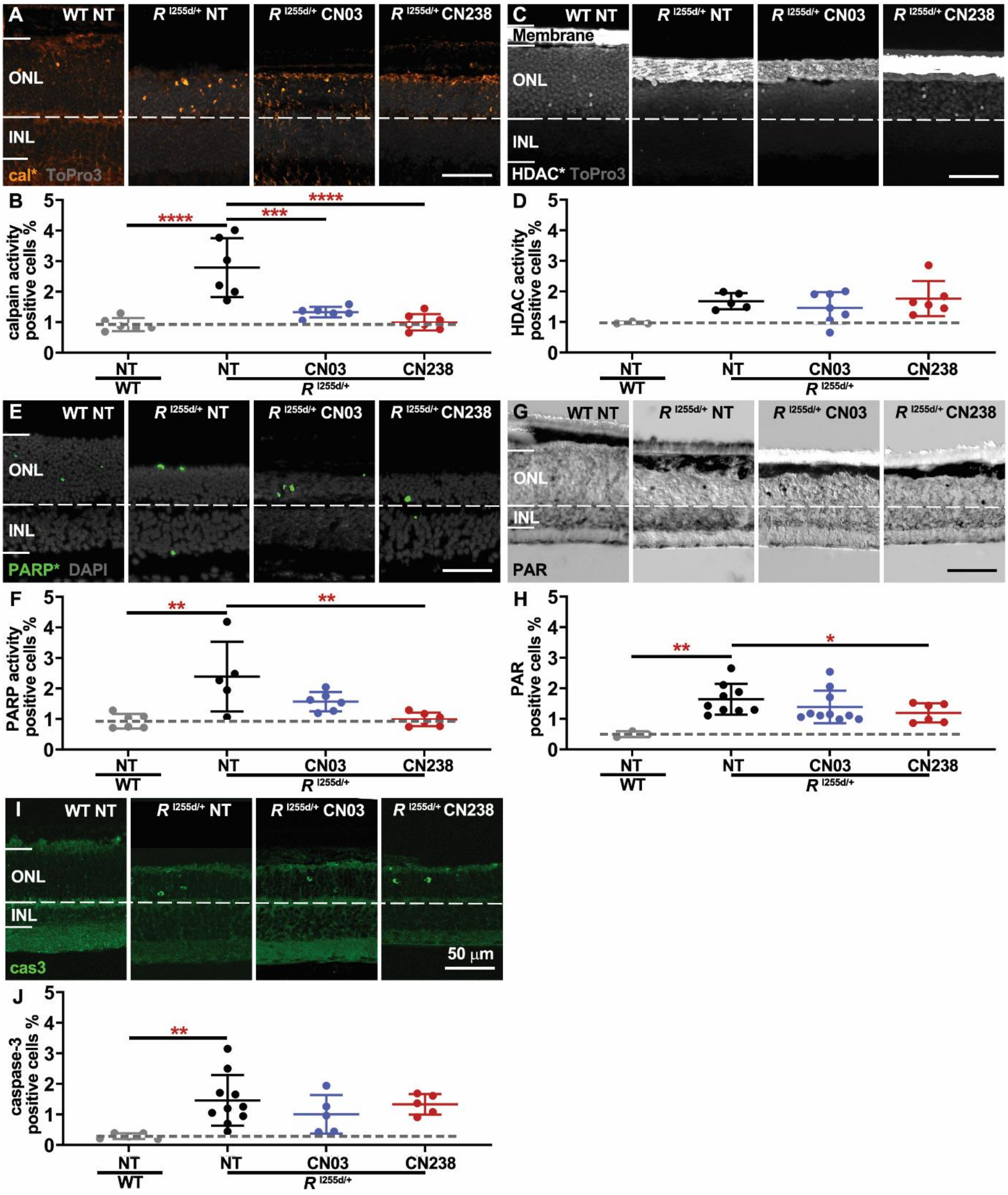

Figure S2. cGMP analogues reduce photoreceptor cell death without decreasing apoptosis. Organotypic retinal explants were obtained from *Rho*$^{I255d/+}$ and wild-type (WT) mice at post-natal (P) day 12 and were treated with cGMP analogues from P14 to P20. **A.** Staining for *in situ* calpain activity (orange). **B.** Percentage of calpain-activity positive cells in the outer nuclear layer (ONL). In *Rho*$^{I255d/+}$, CN03 and CN238 decreased the percentage of calpain-activity-

positive cells compared to the non-treated (NT) situation. **C.** Staining for *in situ* HDAC activity (white). **D.** Quantification of histone deacetylase (HDAC) -activity-positive cells in the ONL showed no significant difference between NT and treatments. **E.** Staining for *in situ* poly (ADP-ribose) polymerase (PARP) activity (green). **F.** Quantification of PARP-activity-positive cells in the ONL revealed an increased percentage in NT *Rho*$^{I255d/+}$ compared to WT. Treatment with CN238 significantly decreased PARP activity in *Rho*$^{I255d/+}$ mutant retina, whereas CN03 had no effect. **G.** Staining for poly (ADP-ribose) (PAR; black). **H.** Quantification of PAR-labeled cells showed an increase in mutant NT retinas compared to WT. CN238 decreased the number of PAR-positive cells in *Rho*$^{I255d/+}$ mutant retina. **I.** Immunostaining (green) for cleaved caspase-3 (cas3). **J.** The percentage of caspase-3-positive cells was elevated in NT mutant compared with NT WT. cGMP analogues had no effect on the numbers of ONL caspase-3 positive cells. DAPI and ToPro3 (both in grey) were used for nuclear counterstaining. n = 3-10 retinas from different animals; error bars indicate SD; statistical testing: Two-way ANOVA with Dunnett's multiple comparisons test; significance level: * $p \le 0.05$, ** $p \le 0.01$, *** $p \le 0.001$, **** $p \le 0.0001$; INL = inner nuclear layer; scale bar = 50 µm.

Figure S3:

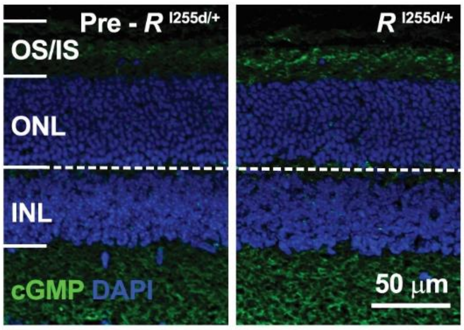

Figure S3. Comparison of pre-absorbed cGMP and standard cGMP immunostaining in *Rho*$^{I255d/+}$ retina. At post-natal day (P) 20 of *Rho*$^{I255d/+}$ retina, immunostaining with the cGMP antibody pre-absorbed with 10 mM cGMP (negative control) showed negligible labeling in the photoreceptor outer and inner segments (OS/IS). In contrast, standard cGMP immunostaining (green), without pre-absorption, distinctly labeled the OS/IS. DAPI (grey) was used as nuclear counterstain. INL = Inner nuclear layer; scale bar = 50 µm.

Supplemental Tables

Table S1. Comparison of cGMP accumulation in outer and inner segments (OS/IS) and outer nuclear layer (ONL) of *Rho*$^{I255d/+}$ and *Rho*$^{I255d/I255d}$: Quantitative data for graphs presented in Figures 1C and 1D.

Table S1 (Figure)	Genotype	Mean ± SD (%)	*p* - value (compared with WT)	n
Fig. 1C	$R^{I255d/+}$ neg	3.74 ± 0.34	*p* = 0.3464	3
	WT	3.20 ± 0.16		3
	$R^{I255d/+}$	4.49 ± 0.54	*p* = 0.0153	3
	$R^{I255d/I255d}$	5.39 ± 0.55	*p* = 0.0006	3
Fig. 1D	$R^{I255d/+}$ neg	51.44 ± 7.19	*p* = 0.1503	3
	WT	142.36 ± 41.26		3
	$R^{I255d/+}$	189.70 ± 64.47	*p* = 0.5746	3
	$R^{I255d/I255d}$	387.61 ± 70.48	*p* = 0.0011	3

Table S2. cGMP accumulation in segments of *Rho*$^{I255d/+}$ and *Rho*$^{I255d/I255d}$: Quantitative data for graph presented in Figure 1E.

Table S2 (Fig. 1E)	Genotype	Peak value (AU)	n
	$R^{I255d/I255d}$	1174.93	3
	$R^{I255d/+}$	585.67	3
	WT	237.87	3
	$R^{I255d/+}$ neg	183.38	3

Table S3. Elevated calpain-2 activation (calpain-2 staining) related to photoreceptor cell death (TUNEL assay) in *Rho*$^{I255d/+}$ retina: Quantitative data for graphs presented in Figures 2B and 2D.

Table S3 (Figure)	Genotype	Mean ± SD (%)	*p* - value (compared with WT)	n
Fig. 2B	WT	0.18 ± 0.06		3
	$R^{I255d/+}$	8.38 ± 1.98	*p* = 0.0020	3
Fig. 2D	WT	0.04 ± 0.01		3
	$R^{I255d/+}$	3.56 ± 1.24	*p* = 0.0079	3

Table S4. Effect of cGMP analogues on photoreceptor cell death (TUNEL assay) in treatments lasting 4-14 days: Quantitative data for graphs presented in Figures 3B, E, H, K.

Table S4 (Figure)	Culture scheme	*p* - value comparison	Genotype - treatment	Mean ± SD (%)	*p* - value	n
Fig. 3B	P12 - 18	$R^{I255d/+}$ - NT	WT - NT	1.73 ± 0.67	$p < 0.0001$	5
			$R^{I255d/+}$ - NT	10.21 ± 3.87		9
			$R^{I255d/+}$ - CN03	5.85 ± 1.38	$p = 0.0007$	10
			$R^{I255d/+}$ - CN238	3.03 ± 0.45	$p < 0.0001$	6
		$R^{I255d/+}$ - CN03	$R^{I255d/+}$ - CN238		$p = 0.0823$	
Fig. 3E	P12 - 20	$R^{I255d/+}$ - NT	WT - NT	3.04 ± 0.26	$p = 0.0011$	3
			$R^{I255d/+}$ - NT	9.94 ± 3.48		9
			$R^{I255d/+}$ - CN03	6.29 ± 2.29	$p = 0.0017$	8
			$R^{I255d/+}$ - CN238	5.02 ± 1.07	$p = 0.0002$	11
		$R^{I255d/+}$ - CN03	$R^{I255d/+}$ - CN238		$p = 0.3106$	
Fig. 3H	P12 - 24	$R^{I255d/+}$ - NT	WT - NT	3.00 ± 1.10	$p < 0.0001$	9
			$R^{I255d/+}$ - NT	7.14 ± 1.79		17
			$R^{I255d/+}$ - CN03	4.40 ± 1.29	$p < 0.0001$	7
			$R^{I255d/+}$ - CN238	4.68 ± 1.08	$p < 0.0001$	17
		$R^{I255d/+}$ - CN03	$R^{I255d/+}$ - CN238		$p = 0.2473$	
Fig. 3K	P12 - 28	$R^{I255d/+}$ - NT	WT - NT	3.17 ± 0.74	$p < 0.0001$	9
			$R^{I255d/+}$ - NT	7.11 ± 1.78		8
			$R^{I255d/+}$ - CN03	3.93 ± 1.56	$p = 0.0001$	12
			$R^{I255d/+}$ - CN238	4.53 ± 0.86	$p = 0.0009$	12
		$R^{I255d/+}$ - CN03	$R^{I255d/+}$ - CN238		$p = 0.2997$	

Table S5. Effect of cGMP analogues on photoreceptor viability (ONL row counts) in treatments lasting 4-14 days: Quantitative data for graphs presented in Figures 3C, F, I, L.

Table S5 (Figure)	Culture scheme	*p* - value comparison	Genotype - treatment	Mean ± SD (%)	*p* - value	n
Fig. 3C	P12 - 18	$R^{I255d/+}$ - NT	WT - NT	10.76 ± 0.41	$p = 0.0004$	5
			$R^{I255d/+}$ - NT	7.70 ± 1.53		9
			$R^{I255d/+}$ - CN03	7.47 ± 0.97	$p = 0.8376$	10
			$R^{I255d/+}$ - CN238	7.41 ± 0.53	$p = 0.2428$	6
		$R^{I255d/+}$ - CN03	$R^{I255d/+}$ - CN238		$p = 0.0823$	
Fig. 3F	P12 - 20	$R^{I255d/+}$ - NT	WT - NT	10.07 ± 0.41	$p < 0.0001$	3
			$R^{I255d/+}$ - NT	4.47 ± 1.01		9
			$R^{I255d/+}$ - CN03	5.12 ± 1.23	$p = 0.3398$	8
			$R^{I255d/+}$ - CN238	5.38 ± 0.35	$p = 0.1741$	11
		$R^{I255d/+}$ - CN03	$R^{I255d/+}$ - CN238		$p = 0.5970$	
Fig. 3I	P12 - 24	$R^{I255d/+}$ - NT	WT - NT	7.70 ± 0.34	$p < 0.0001$	9
			$R^{I255d/+}$ - NT	3.66 ± 1.97		17
			$R^{I255d/+}$ - CN03	2.77 ± 0.74	$p = 0.1042$	7
			$R^{I255d/+}$ - CN238	5.45 ± 2.08	$p < 0.0001$	17
		$R^{I255d/+}$ - CN03	$R^{I255d/+}$ - CN238		$p = 0.1042$	
Fig. 3L	P12 - 28	$R^{I255d/+}$ - NT	WT - NT	7.02 ± 0.70	$p < 0.0001$	9
			$R^{I255d/+}$ - NT	1.39 ± 0.49		8
			$R^{I255d/+}$ - CN03	1.55 ± 0.55	$p = 0.3265$	12
			$R^{I255d/+}$ - CN238	2.69 ± 1.35	$p = 0.0005$	12
		$R^{I255d/+}$ - CN03	$R^{I255d/+}$ - CN238		$p = 0.0010$	

Table S6. Effect of cGMP analogues on calpain-2 activation (calpain-2 staining) in treatments lasting 4-14 days: Quantitative data for graphs presented in Figures S1B, D, F, H.

Table S6 (Figure)	Cultured schema	Genotype - treatment	Mean ± SD (%)	*p* - value	n
Fig. S1B	P12 - 18	WT - NT	1.05 ± 0.31	p = 0.0003	5
		$R^{I255d/+}$ - NT	2.93 ± 1.02		9
		$R^{I255d/+}$ - CN03	1.92 ± 0.49	p = 0.0008	10
		$R^{I255d/+}$ - CN238	1.77 ± 0.26	p = 0.0323	6
Fig. S1D	P12 - 20	WT - NT	1.33 ± 0.34	p = 0.0093	3
		$R^{I255d/+}$ - NT	3.33 ± 1.44		9
		$R^{I255d/+}$ - CN03	1.80 ± 0.71	p = 0.0077	8
		$R^{I255d/+}$ - CN238	2.18 ± 0.94	p = 0.0408	6
Fig. S1F	P12 - 24	WT - NT	1.19 ± 0.34	p = 0.1385	9
		$R^{I255d/+}$ - NT	1.83 ± 0.94		12
		$R^{I255d/+}$ - CN03	1.35 ± 0.96	p = 0.3348	7
		$R^{I255d/+}$ - CN238	1.41 ± 0.58	p = 0.3904	12
Fig. S1H	P12 - 28	WT - NT	0.83 ± 0.22	p = 0.0126	8
		$R^{I255d/+}$ - NT	2.17 ± 1.39		8
		$R^{I255d/+}$ - CN03	1.43 ± 0.76	p = 0.0885	14
		$R^{I255d/+}$ - CN238	1.52 ± 0.84	p = 0.1098	12

Table S7. Effect of cGMP analogues on cone photoreceptor survival (cone arrestin-3 staining) in treatments lasting 4-14 days: Quantitative data for graphs presented in Figures 4B, E, H, K.

Table S7 (Figure)	Cultured schema	*p* - value comparison	Genotype - treatment	Mean ± SD (%)	*p* - value	n
Fig. 4B	P12 - 18	$R^{I255d/+}$ - NT	WT - NT	16.46 ± 0.91	*p* = 0.1222	5
			$R^{I255d/+}$ - NT	13.08 ± 1.40		12
			$R^{I255d/+}$ - CN03	15.70 ± 1.02	*p* = 0.0036	10
			$R^{I255d/+}$ - CN238	15.65 ± 1.24	*p* = 0.0205	6
		$R^{I255d/+}$ - CN03	$R^{I255d/+}$ - CN238		*p* = 0.6663	
Fig. 4E	P12 - 20	$R^{I255d/+}$ - NT	WT - NT	14.11 ± 1.35	*p* = 0.0178	6
			$R^{I255d/+}$ - NT	11.19 ± 1.69		7
			$R^{I255d/+}$ - CN03	10.98 ± 1.91	*p* = 0.9370	6
			$R^{I255d/+}$ - CN238	12.82 ± 1.66	*p* = 0.1378	6
		$R^{I255d/+}$ - CN03	$R^{I255d/+}$ - CN238		*p* = 0.1378	
Fig. 4H	P12 - 24	$R^{I255d/+}$ - NT	WT - NT	13.35 ± 1.19	*p* = 0.0040	8
			$R^{I255d/+}$ - NT	9.85 ± 4.81		14
			$R^{I255d/+}$ - CN03	7.19 ± 3.95	*p* = 0.6543	7
			$R^{I255d/+}$ - CN238	13.18 ± 2.65	*p* = 0.0721	17
		$R^{I255d/+}$ - CN03	$R^{I255d/+}$ - CN238		*p* = 0.0721	
Fig. 4K	P12 - 28	$R^{I255d/+}$ - NT	WT - NT	12.09 ± 1.42	*p* < 0.0001	9
			$R^{I255d/+}$ - NT	3.29 ± 3.50		7
			$R^{I255d/+}$ - CN03	3.86 ± 2.53	*p* = 0.4541	12
			$R^{I255d/+}$ - CN238	9.97 ± 2.13	*p* < 0.0001	13
		$R^{I255d/+}$ - CN03	$R^{I255d/+}$ - CN238		*p* < 0.0001	

Table S8. Effect of cGMP analogues on length of cone photoreceptor outer and inner segments (cone arrestin-3 staining) in treatments lasting 4-14 days: Quantitative data for graphs presented in Figures 4C, F, I, L.

Table S8 (Figure)	Cultured schema	*p* - value comparison	Genotype - treatment	Mean ± SD (%)	*p* - value	n
Fig. 4C	P12 - 18	$R^{I255d/+}$ - NT	WT - NT	10.50 ± 0.89	$p < 0.0001$	5
			$R^{I255d/+}$ - NT	4.53 ± 0.67		6
			$R^{I255d/+}$ - CN03	5.64 ± 1.02	$p = 0.0870$	6
			$R^{I255d/+}$ - CN238	6.20 ± 0.39	$p = 0.0147$	6
		$R^{I255d/+}$ - CN03	$R^{I255d/+}$ - CN238		$p = 0.2795$	
Fig. 4F	P12 - 20	$R^{I255d/+}$ - NT	WT - NT	9.36 ± 0.63	$p < 0.0001$	5
			$R^{I255d/+}$ - NT	4.19 ± 0.59		7
			$R^{I255d/+}$ - CN03	4.91 ± 0.49	$p = 0.0327$	6
			$R^{I255d/+}$ - CN238	6.05 ± 0.53	$p = 0.0005$	6
		$R^{I255d/+}$ - CN03	$R^{I255d/+}$ - CN238		$p = 0.0327$	
Fig. 4I	P12 - 24	$R^{I255d/+}$ - NT	WT - NT	7.54 ± 0.68	$p < 0.0001$	6
			$R^{I255d/+}$ - NT	3.81 ± 0.74		6
			$R^{I255d/+}$ - CN03	3.62 ± 0.49	$p = 0.6222$	6
			$R^{I255d/+}$ - CN238	5.66 ± 0.55	$p = 0.0004$	6
		$R^{I255d/+}$ - CN03	$R^{I255d/+}$ - CN238		$p = 0.0002$	
Fig. 4L	P12 - 28	$R^{I255d/+}$ - NT	WT - NT	7.09 ± 0.84	$p < 0.0001$	6
			$R^{I255d/+}$ - NT	1.20 ± 1.02		6
			$R^{I255d/+}$ - CN03	1.55 ± 0.60	$p = 0.3858$	6
			$R^{I255d/+}$ - CN238	3.21 ± 0.38	$p = 0.0003$	8
		$R^{I255d/+}$ - CN03	$R^{I255d/+}$ - CN238		$p = 0.0010$	

Table S9. Effect of cGMP analogues on calpain, HDAC, and PARP activity, PAR accumulation, after 6-day treatment: Quantitative data for graphs presented in Figures S2B, D, F, H, J.

Table S9 (Figure)	Genotype - treatment	Mean ± SD (%)	*p* - value (compared with $R^{I255d/+}$ - NT)	n
Fig. S2B calpain activity	WT - NT	0.92 ± 0.22	$p < 0.0001$	6
	$R^{I255d/+}$ - NT	2.79 ± 0.96		6
	$R^{I255d/+}$ - CN03	1.33 ± 0.17	$p = 0.0004$	6
	$R^{I255d/+}$ - CN238	0.99 ± 0.27	$p < 0.0001$	7
Fig. S2D HDAC activity	WT - NT	0.97 ± 0.04	$p = 0.1893$	3
	$R^{I255d/+}$ - NT	1.68 ± 0.27		5
	$R^{I255d/+}$ - CN03	1.47 ± 0.51	$p = 0.9487$	7
	$R^{I255d/+}$ - CN238	1.76 ± 0.57	$p = 0.9771$	6
Fig. S2F PARP activity	WT - NT	0.93 ± 0.24	$p = 0.0027$	6
	$R^{I255d/+}$ - NT	2.39 ± 1.14		5
	$R^{I255d/+}$ - CN03	1.57 ± 0.32	$p = 0.0890$	6
	$R^{I255d/+}$ - CN238	0.99 ± 0.22	$p = 0.0037$	6
Fig. S2H PAR accumulation	WT - NT	0.50 ± 0.10	$p = 0.0019$	3
	$R^{I255d/+}$ - NT	1.64 ± 0.50		9
	$R^{I255d/+}$ - CN03	1.39 ± 0.53	$p = 0.6181$	10
	$R^{I255d/+}$ - CN238	1.19 ± 0.32	$p = 0.0478$	6
Fig. S2J cleaved caspase-3	WT - NT	0.29 ± 0.09	$p = 0.0039$	5
	$R^{I255d/+}$ - NT	1.46 ± 0.83		10
	$R^{I255d/+}$ - CN03	1.01 ± 0.63	$p = 0.1067$	5
	$R^{I255d/+}$ - CN238	1.33 ± 0.33	$p = 0.3975$	5
